# Virulent secondary metabolites of entomopathogenic bacteria genera, *Xenorhabdus* and *Photorhabdus*, inhibit phospholipase A_2_ to suppress host insect immunity

**DOI:** 10.1186/s12866-020-02042-9

**Published:** 2020-11-23

**Authors:** Md. Mahi Imam Mollah, Yonggyun Kim

**Affiliations:** Department of Plant Medicals, College of Life Sciences, Andong National University, Andong, 36729 South Korea

**Keywords:** *Xenorhabdus*, *Photorhabdus*, Secondary metabolite, Pathogenicity, Eicosanoid, Immunity

## Abstract

**Background:**

*Xenorhabdus* and *Photorhabdus* are entomopathogenic bacteria that cause septicemia and toxemia in insects. They produce secondary metabolites to induce host immunosuppression. Their metabolite compositions vary among bacterial species. Little is known about the relationship between metabolite compositions and the bacterial pathogenicity. The objective of this study was to compare pathogenicity and production of secondary metabolites of 14 bacterial isolates (species or strains) of *Xenorhabdus* and *Photorhabdus*.

**Results:**

All bacterial isolates exhibited insecticidal activities after hemocoelic injection to *Spodoptera exigua* (a lepidopteran insect) larvae, with median lethal doses ranging from 168.8 to 641.3 CFU per larva. Bacterial infection also led to immunosuppression by inhibiting eicosanoid biosynthesis. Bacterial culture broth was fractionated into four different organic extracts. All four organic extracts of each bacterial species exhibited insecticidal activities and resulted in immunosuppression. These organic extracts were subjected to GC-MS analysis which predicted 182 compounds, showing differential compositions for 14 bacteria isolates. There were positive correlations between total number of secondary metabolites produced by each bacterial culture broth and its bacterial pathogenicity based on immunosuppression and insecticidal activity. From these correlation results, 70 virulent compounds were selected from secondary metabolites of high virulent bacterial isolates by deducting those of low virulent bacterial isolates. These selected virulent compounds exhibited significant immunosuppressive activities by inhibiting eicosanoid biosynthesis. They also exhibited relatively high insecticidal activities.

**Conclusion:**

Virulence variation between *Xenorhabdus* and *Photorhabdus* is determined by their different compositions of secondary metabolites, of which PLA_2_ inhibitors play a crucial role.

**Supplementary Information:**

The online version contains supplementary material available at 10.1186/s12866-020-02042-9.

## Background

*Xenorhabdus* and *Photorhabdus* are entomopathogenic bacteria that exhibit mutualistic symbiosis with entomopathogenic nematodes *Steinernema* and *Heterorhabditis*, respectively [1]. These nematodes can carry and release these bacteria into insect hemocoel by infecting susceptible insect larvae, in which these bacteria can kill insects and convert the cadaver into a food source suitable for nematode growth and development [2]. When nematode population increases to a certain carrying level in a specific insect host, these nematodes are re-associated with specific symbiotic bacteria before emerging from the insect cadaver to search for a new host [3]. There are complex chemical communications in tripartite interactions of bacteria-nematode for symbiosis, bacteria-insect for pathogenicity, and nematode-insect for host recognition. Pathogenic interactions between bacteria and susceptible insects have been relatively well studied regarding the production of specialized metabolites derived from non-ribosomal peptide synthetase (NRPS) or polyketide synthase (PKS) [4]. However, there are secondary compounds other than NRPS-PKS produced from synthetic machineries of bacteria [5].

Virulence of entomopathogenic bacteria exhibits variations among species and strains [6, 7]. Bacteria can secrete several virulence factors in insect hemocoel to suppress insect immune responses and cause fatal septicemia [8, 9]. To induce immunosuppression, both bacterial genera commonly inhibit phospholipase A_2_ (PLA_2_) activity of insects [10]. PLA_2_ is known to catalyze the release of arachidonic acid from phospholipids, which is a committed step to produce various eicosanoids [11]. Eicosanoids mediate cellular and humoral immune responses against various microbial pathogens in insects [12]. Indeed, *X. nematophila* can secrete at least eight secondary metabolites to suppress insect immunity by inhibiting PLA_2_ [13]. Inter-specific variations in *Xenorhabdus* bacterial virulence have been explained by variations in their inhibitory activities against PLA_2_ activities [14]. Intra-specific variations in bacterial virulence of *X. nematophila* have also been reported and explained by differences in immunosuppression due to differential inhibitory effects on PLA_2_ catalytic activity [15]. Park et al. [16] have explained that different virulence due to phase variation is associated with the expression of a specific outer membrane protein. This discovery on virulence gene is further extended by the finding that the expression level of leucine-responsive protein (*Lrp*), a global transcriptional factor, can modulate bacterial pathogenicity [17]. Expression levels of *Lrp* and other transcriptional factors can modulate secondary metabolite production [18], suggesting that the production of secondary metabolites might be positively correlated with bacterial pathogenicity.

Secondary metabolites produced by NRPS and PKS of different species of *Xenorhabdus* and *Photorhabdus* are different in their compositions [19]. Different secondary metabolites can inhibit diverse physiological molecules of susceptible insects to induce immunosuppression. For example, rhabducin, an isocyanide-containing compound produced from biosynthetic gene cluster, can inhibit the activity of phenoloxidase (PO) in *Galleria mellonella* [20]. More than 70 kinds of rhabdopeptide/xenortide peptides derived from NRPS are structurally similar to protease inhibitors. They might degrade various proteins associated with immunity [21, 22]. Phurealipids produced from NRPS/PKS can prevent the expression of antimicrobial peptide genes [23]. Thus, diverse secondary metabolites produced by entomopathogenic bacteria might effectively suppress insect immune responses to induce septicemia.

The aim of this study was to determine virulent secondary metabolites produced by *Xenorhabdus* or *Photorhabdus* based on their inhibitory activities against insect PLA_2_. To this end, this study compared virulence and secondary metabolites of 14 different bacterial isolates of *Xenorhabdus* and *Photorhabdus.*

## Results

### Variations in bacterial virulence

Insecticidal activities of 14 bacterial isolates (species or strains) of *Xenorhabdus* and *Photorhabdus* were assessed by hemocoelic injection of freshly grown live bacteria into L5 larvae of *S. exigua* (Table 1)*.* All bacterial treatments exhibited insecticidal activities. However, their insecticidal activities were different, with LD_50_ ranging from 168.8 (*P. temperata temperata*: ‘Ptt’) to 641.3 (*X. ehlersii*: ‘Xe’) CFU/larva.

**Table 1:** Insecticidal activities of 14 entomopathogenic bacteria (EPB) of *Xenorhabdus* and *Photorhabdus* against L5 larvae of *S. exigua.* Larvae were hemocoelically injected with different doses of freshly cultured bacteria. Before injection, larvae were surface sterilized with 70% ethanol. For each test dose, 10 larvae were used with three replications

EPB^a^	LD_50_ (cfu/larva) (95% CI)	Slope ± SE	df	χ2
Ptt	168.8 (110.4–318.3)	0.87 ± 0.27	1	0.817
Xh	179.2 (156.9–345.6)	0.77 ± 0.29	2	0.953
Xn F	216.9 (114.6–406.8)	0.82 ± 0.27	2	0.906
Xb	222.4 (120.3–432.7)	0.77 ± 0.28	2	0.835
Xn M	244.3 (147.5–480.4)	0.87 ± 0.26	2	0.959
Pl 193	244.3 (128.6–487.3)	0.87 ± 0.26	2	0.959
Pl laum	274.8 (150.3–512.6)	0.74 ± 0.29	2	0.985
Xn SK2	274.9 (151.4–523.2)	0.74 ± 0.29	2	0.985
Pl thra	282.87 (148.8–551.4)	0.97 ± 0.24	2	0.911
Xn 12,145	360.7 (201.7–680.4)	0.85 ± 0.26	2	0.849
Xn K1	550.9 (296.8–1079.8)	0.64 ± 0.32	1	0.879
Xn SK1	552.3 (294.2–1089.5)	0.64 ± 0.32	2	0.987
Xp	556.9 (289.5–1069.5)	0.64 ± 0.32	1	0.784
Xe	641.3 (346.4–1192.7)	0.66 ± 0.31	1	0.964

^a^EPBs include *Photorhabdus temperata* Ss*p. temperata* ANU101 (‘Ptt’), *Xenorhabdus hominickii* ANU101 (‘Xh’), *X. nematophila* France (‘XnF’), *X. bovienii* (‘Xb’), *X. nematophila* Mexico (‘XnM’), *Photorhabdus luminescens* KACC12123 (‘Pl 193’), *P. luminescens* subsp. *laumondii* KACC12283 (‘Pl laum’), *X. nematophila* SK2 (‘XnSK2’), *P. luminescens* subsp. *thracensis* KACC12284 (‘Pl thra’), *X. nematophila* KACC12145 (‘Xn12145’), *Xenorhabdus nematophila* K1 (‘XnK1’), *X. poinarii* (‘Xp’), and *X. ehlersii* KSY (‘Xe’)

**Fig. 1:**
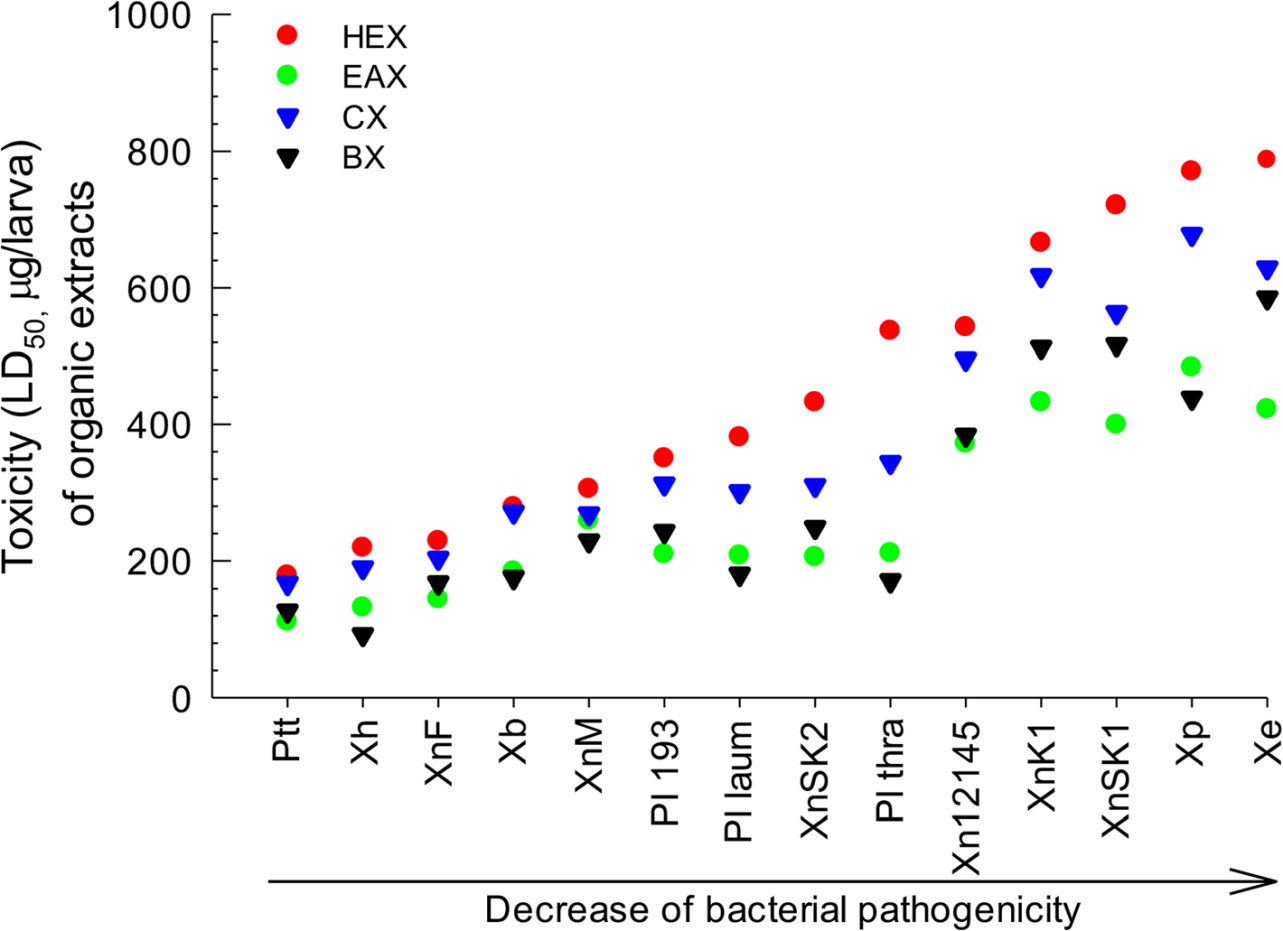
Correlations between insecticidal activities of bacteria and their organic extracts. Bacteria used in this assay were: *Photorhabdus temperata* Subs*p. temperata* ANU101 (‘Ptt’), *Xenorhabdus hominickii* ANU101 (‘Xh’), *X. nematophila* K1 (‘XnK1’), *X. ehlersii* KSY (‘Xe’),, *X. nematophila* SK1 (‘XnSK1’), *X. nematophila* SK2 (‘XnSK2’), *Photorhabdus luminescens* KACC12123 (‘Pl 193’), *P. luminescens* subsp. *laumondii* KACC12283 (‘Pl laum’), *P. luminescens* subsp. *thracensis* KACC12284 (‘Pl thra’), *X. nematophila* KACC12145 (‘Xn12145’), *X. nematophila* Mexico (‘XnM’), *X. nematophila* France (‘XnF’), *X. bovienii* (‘Xb’), and *X. poinarii* (‘Xp’). Bacterial pathogenicity is presented in Table 1. Their cultured broths were fractionated with four different organic solvents: hexane (‘HEX’), ethyl acetate (‘EAX’), chloroform (‘CX’), and butanol (‘BX’). For each treatment, three replications were performed using 10 larvae per replication

To analyze variations in bacterial insecticidal activities, their secondary metabolites were extracted from bacterial culture broth with four different organic solvents: hexane (‘HEX’), ethyl acetate (‘EAX’), chloroform (‘CX’), and butanol (‘BX’) extracts. These organic extracts showed variations in insecticidal activities, with LD_50_ values ranging from 92.4 (BX from *X. hominickii*: ‘Xh’) to 787.6 (HEX from ‘Xe’) μg/larva (Table S1). Insecticidal activities of bacteria and their extracts exhibited high positive correlations (Fig. 1): bacterial toxicity with HEX (*r* = 0.954; *P* < 0.0001), EAX (*r* = 0.938; *P* < 0.0001), CX (*r* = 0.967; *P* < 0.0001), and BX (*r* = 0.967; *P* < 0.0001).

### Comparative analysis of bacterial extracts for insect immunosuppression

**Fig. 2:**
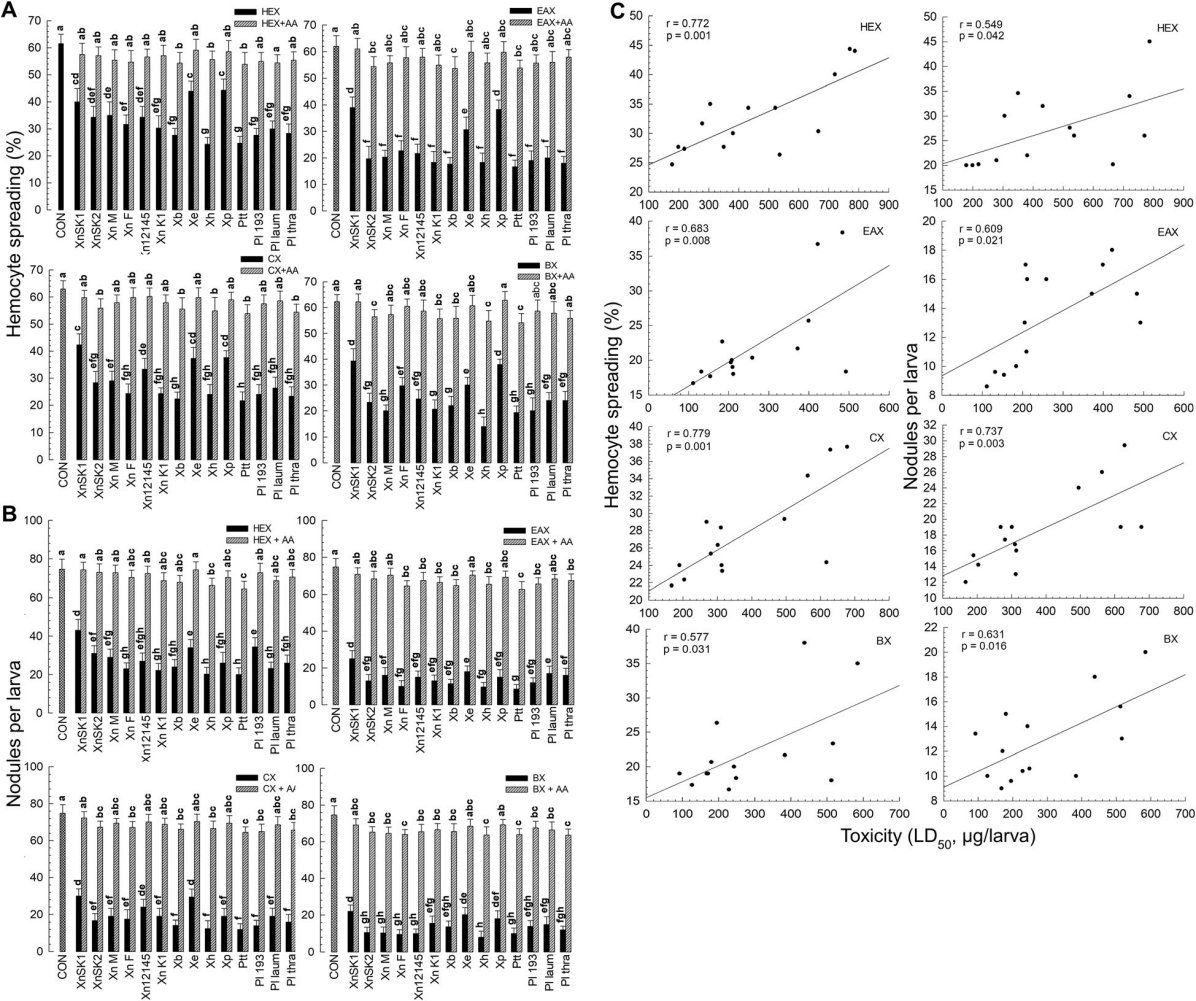
Suppression of cellular immune responses by four organic extracts of 14 bacterial isolates and its correlation with bacterial pathogenicity. Bacteria used in this assay were: *Photorhabdus temperata* Subs*p. temperata* ANU101 (‘Ptt’), *Xenorhabdus hominickii* ANU101 (‘Xh’), *X. nematophila* K1 (‘XnK1’), *X. ehlersii* KSY (‘Xe’),, *X. nematophila* SK1 (‘XnSK1’), *X. nematophila* SK2 (‘XnSK2’), *Photorhabdus luminescens* KACC12123 (‘Pl 193’), *P. luminescens* subsp. *laumondii* KACC12283 (‘Pl laum’), *P. luminescens* subsp. *thracensis* KACC12284 (‘Pl thra’), *X. nematophila* KACC12145 (‘Xn12145’), *X. nematophila* Mexico (‘XnM’), *X. nematophila* France (‘XnF’), *X. bovienii* (‘Xb’), and *X. poinarii* (‘Xp’). Their cultured broths were extracted with four different organic solvents: hexane (‘HEX’), ethyl acetate (‘EAX’), chloroform (‘CX’), and butanol (‘BX’). **a** Effects of organic extracts on hemocyte-spreading behavior. For each treatment, three independently prepared hemocyte mixtures were used. To determine the spreading behavior, 100 hemocytes were randomly chosen. **b** Effects of organic extracts on hemocyte nodulation in response to bacterial challenge. Each L5 larva of *S. exigua* was injected with bacterial extract (10 μg/larva) along with heat-killed *E. coli* (4 × 10^4^ cells). For each treatment, three replications were used with five larvae per replication. Arachidonic acid (AA, a catalytic product of PLA_2_) was used to rescue the inhibition. Different letters above standard deviation bars indicate significant differences among means at Type I error = 0.05 (LSD test). **c** Correlations (r) between insecticidal activities of bacterial extracts (Fig. 1) and their immunosuppressive activities. Lines represent the best-fit regression

Variations in insecticidal activities among entomopathogenic bacteria might be caused by their differential immunosuppressive capabilities against target insects. To test this hypothesis, effects of bacterial extracts on cellular immune responses were assessed using hemocyte-spreading behaviour and nodulation assays after bacterial infection. All extracts significantly (*p* < 0.05) inhibited the hemocyte-spreading behavior (Fig. 2a) and nodulation (Fig. 2b). Immunosuppression was significantly (*p* < 0.05) rescued by the addition of arachidonic acid (a catalytic product of PLA_2_), suggesting that the immunosuppression was caused by the inhibition of PLA_2_. However, inhibitory activities of these bacterial extracts were different among bacterial species. When bacterial insecticidal activities were compared with immunosuppressive activities, these two parameters showed high positive correlations for all extracts (Fig. 2c).

**Fig. 3:**
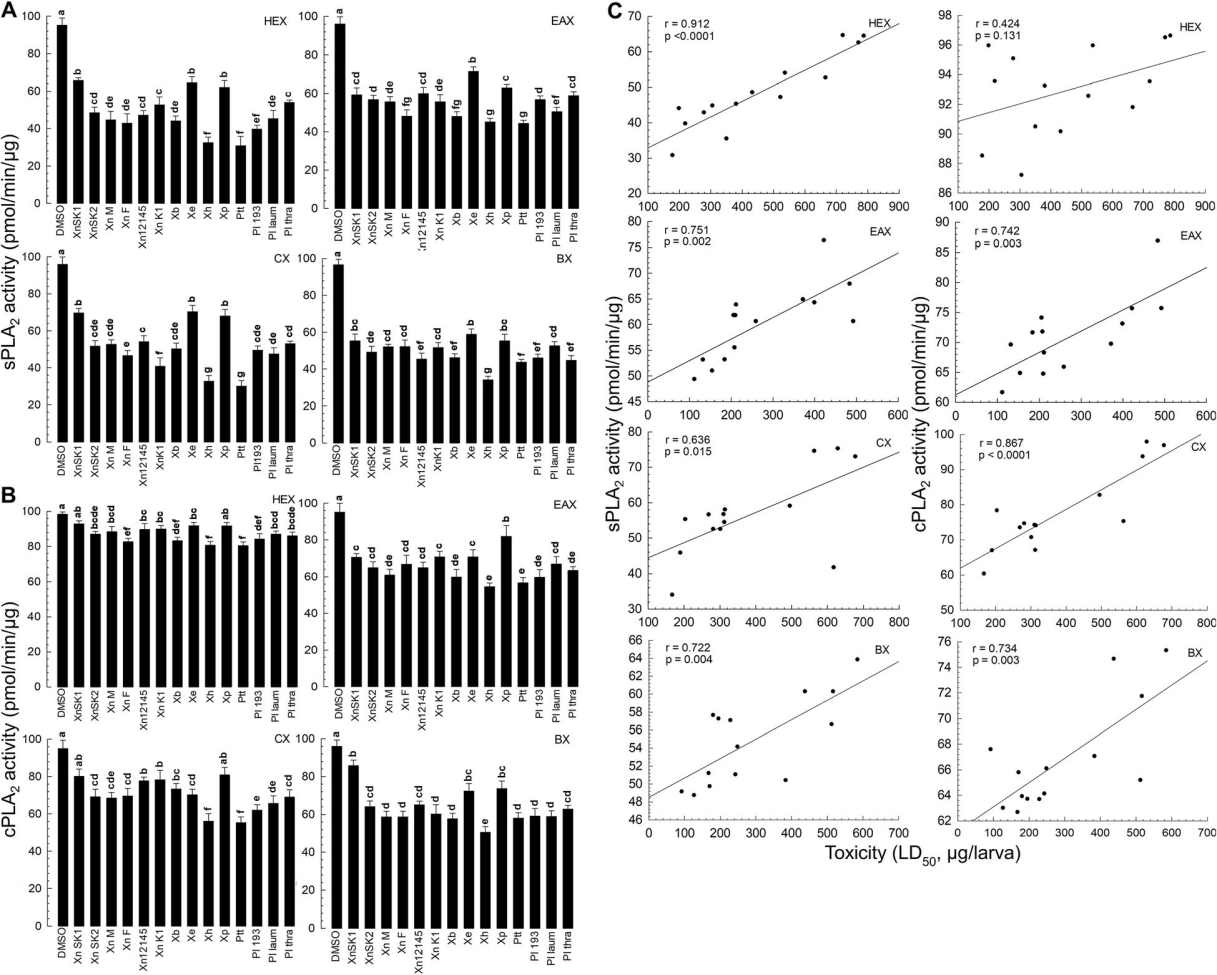
Inhibitory activities of four organic extracts of 14 bacterial isolates against PLA_2_ enzyme and their correlation with bacterial pathogenicity. Bacteria used in this assay included 14 isolates: *Photorhabdus temperata* Subs*p. temperata* ANU101 (‘Ptt’), *Xenorhabdus hominickii* ANU101 (‘Xh’), *X. nematophila* K1 (‘XnK1’), *X. ehlersii* KSY (‘Xe’),, *X. nematophila* SK1 (‘XnSK1’), *X. nematophila* SK2 (‘XnSK2’), *Photorhabdus luminescens* KACC12123 (‘Pl 193’), *P. luminescens* subsp. *laumondii* KACC12283 (‘Pl laum’), *P. luminescens* subsp. *thracensis* KACC12284 (‘Pl thra’), *X. nematophila* KACC12145 (‘Xn12145’), *X. nematophila* Mexico (‘XnM’), *X. nematophila* France (‘XnF’), *X. bovienii* (‘Xb’), and *X. poinarii* (‘Xp’). Their cultured broths were extracted with four different organic solvents: hexane (‘HEX’), ethyl acetate (‘EAX’), chloroform (‘CX’), and butanol (‘BX’). **a** Effects of organic extracts on sPLA_2_. **b** Effects of organic extracts on cPLA_2_. DMSO was used as control. Each treatment was replicated three times using independent samples. Different letters above standard deviation bars indicate significant differences among means at Type I error = 0.05 (LSD test). **c** Correlations (r) between insecticidal activities of bacterial extracts (Fig. 1) and their inhibitory activities against PLA2 enzyme. Lines represent the best-fit regression

To determine whether immunosuppressive activities of these bacterial extracts were caused by inhibition of insect PLA_2_, these extracts were incubated with PLA_2_ extract obtained from *S. exigua* hemocytes. All bacterial extracts significantly inhibited sPLA_2_ activity, although they showed variations in inhibiting sPLA_2_ (Fig. 3a). For cPLA_2_, some extracts failed to inhibit its enzyme activity (Fig. 3b). Compared to HEX and CX, EAX and BX appeared to show higher inhibitory activities. Ptt and Xh extracts showed significantly higher inhibitory activities than the other bacterial extracts. There were positive correlations between bacterial insecticidal activities and PLA_2_ inhibition except HEX against cPLA_2_ (Fig. 3c).

### Prediction of virulent secondary metabolites produced by *Xenorhabdus* and *Photorhabdus*

**Fig. 4:**
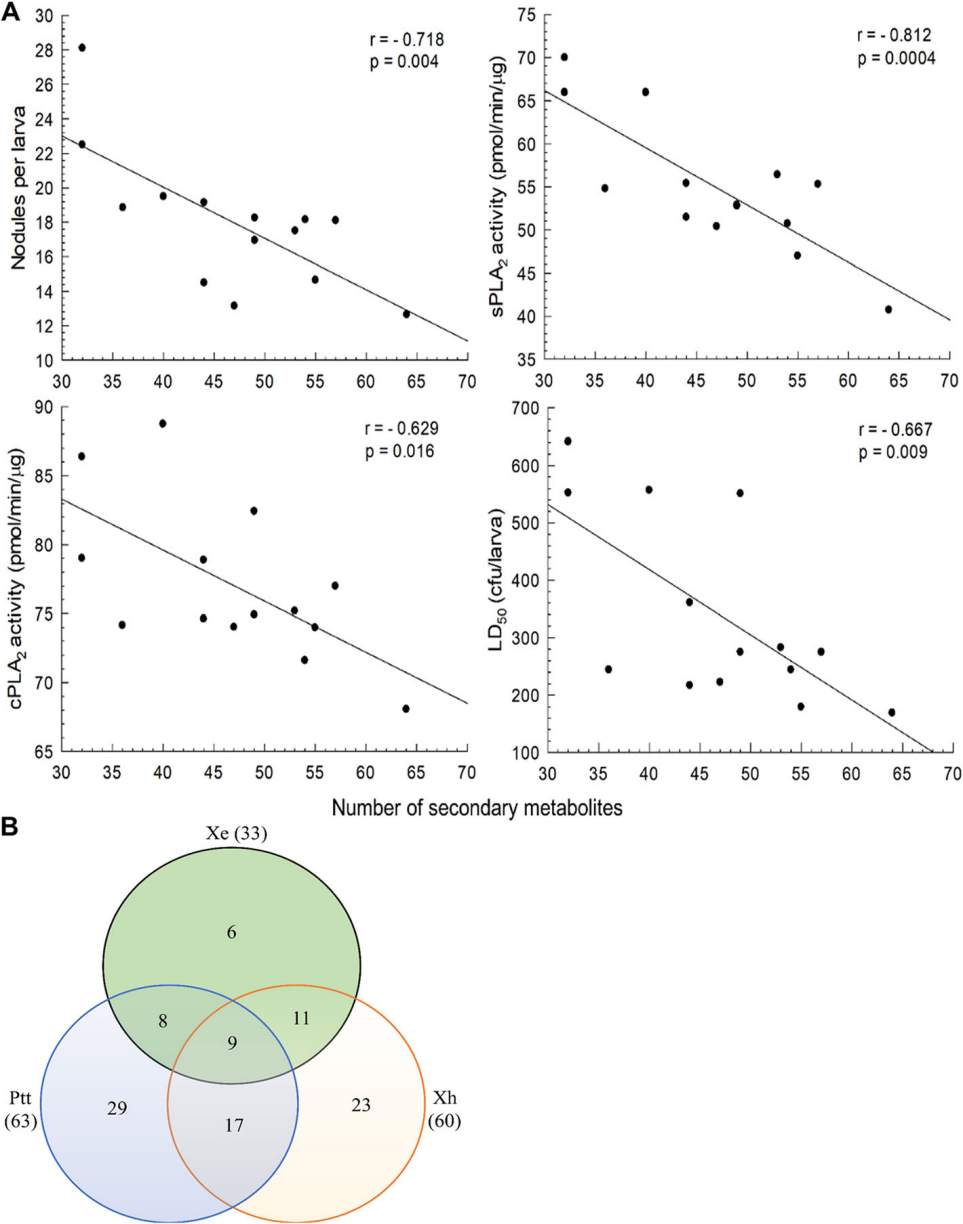
Prediction of virulent secondary metabolites produced by *Xenorhabdus* and *Photorhabdus*. **a** Correlations (r) between total number of secondary metabolites and virulent parameters of hemocyte nodulation suppression, inhibition of sPLA_2_ or cPLA_2_, and insecticidal activities (LD_50_) of 14 bacterial isolates (**b**) Venn diagram analysis of secondary metabolites produced by *Photorhabdus temperata* Subs*p. temperata* (‘Ptt’), *Xenorhabdus hominickii* (‘Xh’), and *X. ehlersii* (‘Xe’). Figures in parentheses indicate total numbers of bacterial secondary metabolites predicted by GC-MS

Immunosuppressive activities of bacterial extracts suggested that their secondary metabolites contained inhibitory compounds. To predict functional secondary metabolites, all extracts were assessed by TLC to confirm the presence of compounds. These compounds were then analyzed by GC-MS (Figure S1). Predicted compounds of four extracts for each bacterial species were combined and resulting 182 compounds were compared between bacterial isolates (Table S2). In addition to different compositions among bacterial isolates, the total number of secondary metabolites varied from 32 compounds of *X. nematophila* SK1 to 63 compounds of Ptt. Interestingly, there was a significant (*p* < 0.05) negative correlation between the total number of secondary metabolites and target insect immune responses measured by nodulation, sPLA_2_ activity, or cPLA_2_ (Fig. 4a). In addition, if bacteria had more secondary metabolites, they exhibited higher insecticidal activities.

To select virulent bacterial compounds, two most potent bacteria, Ptt and Xh, were compared with the least potent Xe for their secondary metabolites (Fig. 4b). Ptt and Xh shared over 44% metabolites. Almost 82% metabolites of Xe were detected in the culture broth of Ptt or Xh. Thus, Ptt- (29 compounds) or Xh- (23 compounds) specific compounds and overlapping (17 compounds) compounds were chosen as possible virulent compounds (Table 2).

**Table 2:** Prediction of 70 virulent secondary metabolites derived from *X. hominickii* (‘Xh’) and *P. temperata temperata* (‘Ptt’). These metabolites were predicted from GC-MS analysis of extracts of bacterial culture broths

Group^a^	No	Compounds
Ptt + Xh	1	1-Butanamine, N-butyl-
	2	Benzyl alcohol
	3	5-Thiazoleethanol, 4-methyl-
	4	Indole
	5	Benzeneethanol, 4-hydroxy-
	6	Phthalimide
	7	o-Cyanobenzoic acid
	8	Acetamide, N-(2-phenylethyl)-
	9	Indole-3-pyruvic acid
	10	Tryptophol
	11	1H-Indole-3-acetic acid, hydrazide
	12	2-Mercaptophenol
	13	Pyrrolo[1,2-a] pyrazine-1,4-dione, hexahydro-
	14	1H-Indole-3-ethanol, acetate (ester)
	15	2-Dodecen-1-yl (−) succinic anhydride
	16	Pyrrolo[1,2-a] pyrazine-1,4-dione, hexahydro-3-(2-methylpropyl)-
	17	2,5-Piperazinedione, 3-(phenylmethyl)-
Xh	1	1-Hexanol, 2-ethyl-
	2	Pyrazine, 3-ethyl-2,5-dimethyl-
	3	Benzeneethanamine
	4	Hexanoic acid, 5-oxo-, ethyl ester
	5	1,1-Diisobutoxy-isobutane
	6	Formamide, N, N-dibutyl-
	7	Cyclohexasiloxane, dodecamethyl-
	8	Butanoic acid, butyl ester
	9	Propanoic acid, 2-methyl-, butyl ester
	10	2-Tetradecene, (E)-
	11	3-Ethoxy-4-Methoxyphenol
	12	7,9-Dimethyl-1,4-dioxa-7,9-diazacycloundecane-8-thione
	13	Heptadecane, 2,6-dimethyl-
	14	1H-Indole-3-acetic acid, methyl ester
	15	1H-Indene, 2-butyl-5-hexyloctahydro-
	16	L-Proline, N-valeryl-, decyl ester
	17	Stannane, tetraethyl-
	18	Fluorene, 4-[1,2-dihydroxyethyl]-
	19	1-Eicosene
	20	Nonadecanenitrile
	21	Heptadecanoic acid, 14-methyl-, methyl ester
	22	E-8-Methyl-9-tetradecen-1-ol acetate
	23	Pentanamide, N-[2-(indol-3-yl)] ethyl-
Ptt	1	4-Ethylamino-n-butylamine
	2	2,5-Dimethyl-4-hydroxy-3(2H)-furanone
	3	Octanoic acid
	4	Benzothiazole
	5	1,2-Ethanediol, 1-phenyl-
	6	n-Decanoic acid
	7	Phenol, 2,6-bis(1,1-dimethylethyl)-
	8	Dodecanoic acid
	9	1-Pentadecene
	10	1H-Benzimidazole, 2-(methylthio)-
	11	Propanamide, 2-amino-3-(3-indolyl)-
	12	Hexadecane, 7,9-dimethyl-
	13	1-Hexadecanol, 2-methyl-
	14	Dicyclohexyldisulphide
	15	1-Nonadecene
	16	Propanamide, 2,2,3,3,3-pentafluoro-N-(2-phenylethyl)-
	17	1,13-Tetradecadien-3-one
	18	Hexadecanoic acid, methyl ester
	19	Diethyl Phthalate
	20	E-15-Heptadecenal
	21	3-Phenylbicyclo (3.2.2) nona-3,6-dien-2-one
	22	E-11-Methyl-12-tetradecen-1-ol acetate
	23	2-Methyl-E-7-octadecene
	24	Octadecanenitrile
	25	Pyrene, 4-methyl-
	26	9-Octadecenamide, (Z)-
	27	Di-n-octyl phthalate
	28	Zinc, bis (dimethylcarbamodithioato-S, S′)-, (T-4)-
	29	Zinc dibutyldithiocarbamate

^a^Groups are classified by bacterial metabolites from both Ptt and Xh (‘Ptt + Xh’), only Xh (‘Xh’), and only Ptt (‘Ptt’)

### Validation of virulent secondary metabolites for their immunosuppression and insecticidal activities

**Fig. 5:**
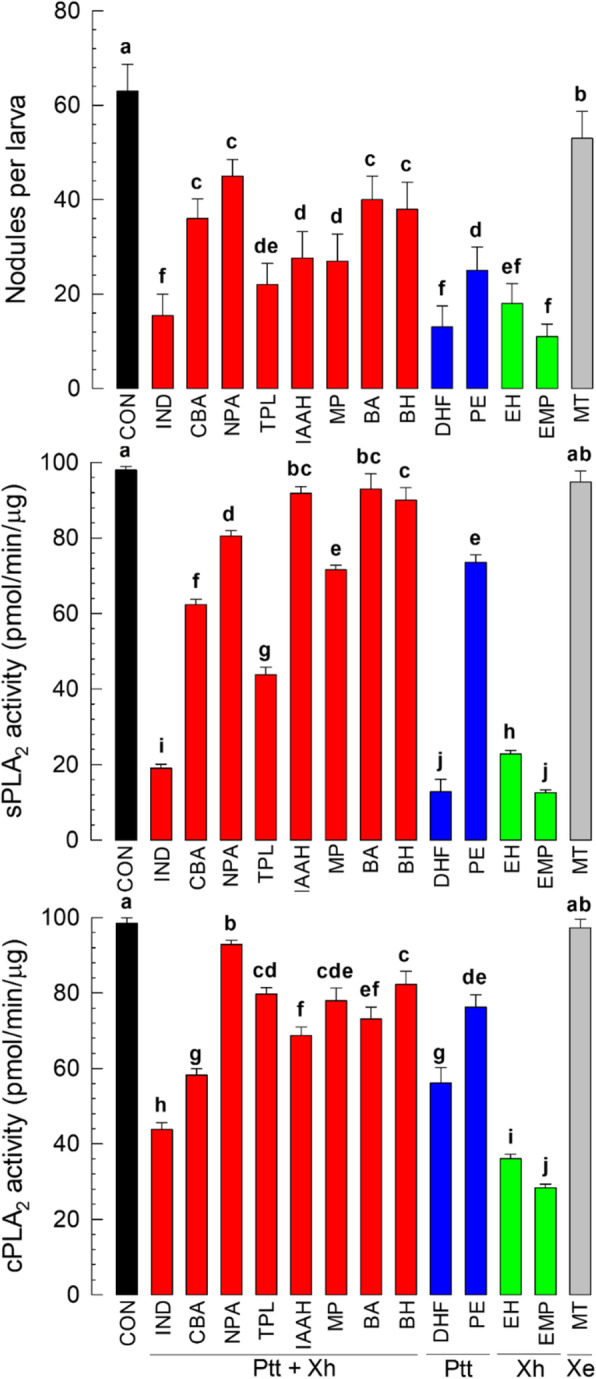
Validation of virulence activities of 12 virulent candidate secondary metabolites produced by two highly potent bacterial isolates of *Photorhabdus temperata* Subs*p. temperata* (‘Ptt’) and *Xenorhabdus hominickii* (‘Xh’). A reference metabolite was selected from compounds produced by a low virulent isolate of *X. ehlersii* (‘Xe’). For virulence tests, suppression of hemocyte nodulation, inhibition of sPLA_2_, and inhibition of cPLA_2_ were determined. To induce hemocyte nodulation, 4 × 10^4^ cfu/larva of *E. coli* was injected to L5 larvae of *S. exigua*. Bacterial metabolites (10 μg/larvae) were injected to inhibit nodule formation. For each treatment, 15 larvae were used. To measure sPLA_2_ and cPLA2 enzyme activities, a commercial assay kit was used with PLA_2_-specific substrate as described in Materials and methods. As control (‘CON’), DMSO was used. Each treatment was independently replicated three times. Different letters above standard error bars indicates significant differences among means at Type I Error = 0.05 (LSD test). Bacterial metabolites used in this assay included benzyl alcohol (‘BA’), benzeneethanol-4-hydroxy (‘BH’), o-cyanobenzoic acid (‘CBA’), 2,5-dimethyl-4-hydroxy-3(2H)-furanone (‘DHF’), 2-ethyl-1-hexanol (‘EH’), 3-ethoxy-4-methoxyphenol (‘EMP’), indole-3-aceticacid hydrazide (‘IAAH’), indole (‘IND’), 2-mercaptophenol (‘MP’), 2-mercaptobenzothiazole (‘MT’), N-(2-phenylethyl) acetamide (‘NPA’), 1-phenyl-1,2-ethanediol (‘PE’), and tryptophol (‘TPL’). These compounds are classified as bacterial metabolites synthesized by both Ptt and Xh (‘Ptt + Xh’), only Xh (‘Xh’), only Ptt (‘Ptt’), and common to Ptt, Xh, and Xe (‘Xe’)

Twelve compounds were selected from 70 virulent candidates produced by Ptt or Xh (Table S3). As a reference, one compound (2-mercaptobenzothiazole: ‘MT’) was randomly selected from metabolites produced by Xe as well as Ptt and Xh. All 12 virulent compounds highly suppressed hemocytic nodulation in response to bacterial infection (Fig. 5a). MT also suppressed immune responses, although it exhibited much lower inhibitory activity than those 12 virulent compounds. Compounds that showed the highest inhibitory activities were indole (‘IND’), ethoxymethoxyphenol (‘EMP’), and dimethylhydroxyfuranone (‘DHF’). These 12 virulent compounds significantly (*p* < 0.05) inhibited sPLA_2_ activity (Fig. 5b) and cPLA_2_ activity (Fig. 5c) whereas MT did not. IND, EMP, and DHF also showed high inhibitory activities against PLA_2_. These 12 virulent compounds also showed high insecticidal activities (Table 3). In contrast, MT had relatively low toxicities to the test insect species. However, IND, EMP, and DHF exhibited high insecticidal activities, with LD_50_ ranging from 4.29 to 5.12 μg/larva.

**Table 3:** Insecticidal activities of virulent secondary metabolites of *P. temperata temperata* (Ptt) and *X. hominickii* (Xh) against *S. exigua*. Virulent metabolites were compared with the reference compound produced by a less virulent *X. ehlersii* (Xe). Bioassays were conducted by hemocoelic injection to L5 larvae. For each treatment dose, 10 larvae were used

Group^a^	Compound^b^	LD_50_ (μg/larva) (95% CI)	Slope ± SE	df	χ2
Ptt + Xh	IND	5.12 (2.51–10.2)	0.50 ± 0.41	3	0.813
	CBA	8.52 (4.2–16.4)	0.47 ± 0.44	3	0.774
	NPA	12.79 (6.1–24.5)	0.32 ± 0.59	3	0.953
	TPL	7.44 (8.8–34.9)	0.44 ± 0.47	3	0.905
	IAAH	14.14 (7.6–27.6)	0.31 ± 0.61	3	0.944
	MP	15.15 (7.9–30.3)	0.29 ± 0.66	3	0.993
	BA	17.93 (9.3–34.7)	0.31 ± 0.63	3	0.940
	BH	22.47 (17.4–62.9)	0.35 ± 0.58	3	0.701
Ptt	DHF	4.94 (3.2–8.7)	0.49 ± 0.42	3	0.718
	PE	24.28 (12.6–47.5)	0.42 ± 0.49	3	0.819
Xh	EH	8.93 (4.8–17.5)	0.43 ± 0.47	3	0.492
	EMP	4.29 (2.1–8.4)	0.36 ± 0.53	3	0.926
Xe	MT	38.75 (20.0–75.6)	0.38 ± 0.54	3	0.691

^a^Group is classified by bacterial compounds specific to (1) both Ptt and Xh (‘Ptt + Xh’), (2) only Ptt (‘Ptt’), (3) only Xh (‘Xh’), and (4) common to Ptt, Xh, and Xe (‘Xe’)

^b^Compound acronyms are: benzyl alcohol (‘BA’), benzeneethanol-4-hydroxy (‘BH’), o-cyanobenzoic acid (‘CBA’), 2,5-dimethyl-4-hydroxy-3(2H)-furanone (‘DHF’), 2-ethyl-1-hexanol (‘EH’), 3-ethoxy-4-methoxyphenol (‘EMP’), indole-3-aceticacid hydrazide (‘IAAH’), indole (‘IND’), 2-mercaptophenol (‘MP’), 2-mercaptobenzothiazole (‘MT’), N-(2-phenylethyl) acetamide (‘NPA’), 1-phenyl-1,2-ethanediol (‘PE’), and tryptophol (‘TPL’)

## Discussion

Bacterial species in genera *Xenorhabdus* and *Photorhabdus* can produce various secondary metabolites to maintain their mutualistic symbiosis with host entomopathogenic nematodes [24]. Some of metabolites are required for their colonization to specific nematode hosts while others are crucial for pathogenesis specifically targeting insect’s immune system [3]. This study assessed all metabolites extracted with four organic solvents from bacterial culture broth of *Xenorhabdus* and *Photorhabdus* to identify their virulent secondary metabolites against insect immune responses.

Organic extracts of bacterial culture broth of seven different species classified in *Xenorhabdus* and *Photorhabdus* showed immunosuppressive and insecticidal activities. However, pathogenic activities of these extracts were different among 14 bacterial isolates and highly correlated with their bacterial insecticidal activities. These results suggest that these bacteria can induce immunosuppression of target insects via their secondary metabolites, supporting the hypothesis that immunosuppression is required for bacterial pathogenicity. Protein toxins that also induce the immunosuppression and finally kill target insects are known in *Xenorhabdus* and *Photorhabdus* [25]. However, acute attack by a target insect’s immune system should be avoided and actively suppressed by the secondary metabolites [26].

Immunosuppression of target insects by *Xenorhabdus* and *Photorhabdus* infection is caused by inhibition of PLA_2_ by bacterial secondary metabolites [10]. The first identified PLA_2_ inhibitor produced by bacteria was benzylideneacetone (BZA) produced by *X. nematophila* [27]. Subsequently, eight different PLA_2_ inhibitors were identified from bacterial culture broths of *X. nematophila* and *P. temperata temperata* [13]. On the other hand, these PLA_2_ inhibitors induced apoptosis-associated cytotoxicity and insecticidal activities [28]. This explains why bacterial metabolites containing PLA_2_ inhibitors in our current study showed insecticidal activities. Indeed, the degree of PLA_2_ inhibition by bacteria has been found to be highly correlated with bacterial pathogenicity [15]. These findings suggested that bacterial metabolites that inhibited PLA_2_ activities in this current study contained virulent secondary metabolites.

Virulent secondary metabolites produced by *Xenorhabdus* and *Photorhabdus* were chosen based on comparative analysis of secondary metabolites among bacterial isolates. GC-MS analyses for bacterial culture broths of 14 isolates predicted 182 compounds. Most of them were phenol derivatives, tryptophan derivatives, peptides, and fatty acid derivatives including secondary metabolites synthesized by NRPS-PKS [4]. In general, predicted and identified compounds from NRPS-PKS of *Xenorhabdus* and *Photorhabdus* genomes are associated with bacterial pathogenicity [19]. This is supported by the high correlation between the number of predicted compounds in each bacterial isolate and bacterial pathogenicity found in the current study. This also suggests that these compounds predicted by GC-MS might contain virulent secondary metabolites. To select virulent compounds, predicted metabolites of highly pathogenic bacterial isolates (Ptt and Xh) were compared with those of a low pathogenic isolate (Xe). The comparison for 103 total compounds resulted in the selection of 70 compounds, including Ptt-specific, Xh-specific, and Ptt + Xh common metabolites. Among 70 virulent candidates, 12 randomly chosen compounds showed high immunosuppressive and insecticidal activities, validating the prediction of these 70 virulent secondary metabolites of *Xenorhabdus* and *Photorhabdus*. This analysis did not include 80 compounds (182 compounds from 14 total isolates – 103 compounds of 3 isolates used for the comparison) because other bacterial isolates were less virulent than Ptt and Xh. Among 12 virulent compounds, three (IND, EMP, and DHF) were highly potent in inhibiting insect immune responses, exhibiting high insecticidal activities. These compounds are not likely to be the secondary metabolites produced from NRPS-PKS. Although secondary metabolites derived from NRPS-PKS contribute to induce the bacterial pathogenicity as mentioned above, non-NRPS-PKS metabolites may also play crucial role in expressing insecticidal activity of the bacteria. This is supported by the fact that a mutant *X. szentirmaii* in a specific phosphopantethienyl transferase, which activates the carrier protein domain of PKS, NRPS, and fatty acid synthase [29], loses the production of NRPS-PKS metabolites but exhibits insecticidal virulence [30]. IND has been found to be a PLA_2_ inhibitor from entomopathogenic bacteria [13]. Unlike tryptophan derivatives including indole, EMP is a phenol derivative that shares structural similarities with other sPLA_2_ inhibitors such as 4-bromophenacyl bromide and BZA. DHF is a furanone and a relatively new chemical compound that can inhibit PLA_2_ among bacterial metabolites. Derivatives containing these three different skeletons need to be compared for their activities to inhibit insect PLA_2_ to develop potent immunosuppressants. In summary, this study newly proposes other PLA_2_ inhibitors including EMP and DHF.

## Conclusions

Bacterial isolates of *Xenorhabdus* and *Photorhabdus* were pathogenic by suppressing insect immune responses via PLA_2_ inhibition using their secondary metabolites. Such immunosuppression was highly correlated with insecticidal activity of bacteria. Based on this correlation, 70 virulent compounds were predicted by comparative analysis of secondary metabolites between high and low pathogenic bacteria.

## Methods

### Insect and bacteria

Larvae of *Spodoptera exigua* were reared at temperature of 25 ± 2 °C and relative humidity of 65 ± 5% under 16 h light and 8 h dark condition with an artificial diet [31]. Under these conditions, larvae underwent five instars (L1-L5). Adults were provided with 10% sucrose solution. For bioassays to determine bacterial pathogenicity, larvae were taken from a cohort.

Ten strains of *Xenorhabdus* and four strains of *Photorhabdus* were collected from previous stocks: [*Xenorhabdus nematophila* K1 (‘XnK1’) [32], *X. hominickii* ANU101 (‘Xh’) [16], *X. ehlersii* KSY (‘Xe’) [33], *Photorhabdus temperata* Ss*p. temperata* ANU101 (‘Ptt’) [34], *X. nematophila* SK1 (‘XnSK1’) and *X. nematophila* SK2 (‘XnSK2’) [15], Korean Agricultural Culture Collection (KACC, RDA, Jeonju, Korea) [*Photorhabdus luminescens* KACC12123 (‘Pl 193’), *P. luminescens* subsp. *laumondii* KACC12283 (‘Pl laum’), *P. luminescens* subsp. *thracensis* KACC12284 (‘Pl thra’), *X. nematophila* KACC12145 (‘Xn12145’), *X. nematophila* Mexico (‘XnM’), *X. nematophila* France (‘XnF’), *X. bovienii* (‘Xb’), and *X. poinarii* (‘Xp’)]. These bacteria were grown in tryptic soy broth (TSB: Difco, Sparks, MD, USA) for 48 h at 28 °C with shaking at 180 rpm [16, 32]. *Escherichia coli* Top 10 was purchased from Invitrogen (Carlsbad, CA, USA) and cultured overnight in Luria-Bertani (LB) medium at 37 °C with shaking at 180 rpm. For immune challenge experiment, heat killed (80 °C for 10 min) *E. coli* were used. Their cell number was counted using a hemocytometer (Neubauer improved bright line, Cat. No. 0640010, Superior Marienfeld, Lauda-Konigshofen, Germany) under a phase contrast microscope (BX41, Olympus, Tokyo, Japan). Different bacterial concentrations were prepared through serial dilution in sterilized deionized distilled water.

### Chemicals

Arachidonic acid (5,8,11,14-eicosatetraenoic acid; AA), tryptophol (TPL), indole (IND), indole-3-aceticacid hydrazide (IAAH), o-cyanobenzoic acid (CBA), 2-ethyl-1-hexanol (EH), 2-mercaptophenol (MP), and 2-mercaptobenzothiazole (MT) were purchased from Sigma-Aldrich Korea (Seoul, Korea) and dissolved in dimethyl sulfoxide (DMSO). 1,2-bis (heptanoylthio) phosphatidylcholine (PC) was used as secretory PLA_2_ (sPLA_2_) substrate while arachidonoyl thio-PC was used as cellular PLA_2_ (cPLA_2_) substrate. These substrates were purchased from Cayman Chemical (Ann Arbor, MI, USA). Benzyl alcohol (BA) and benzeneethanol-4-hydroxy (BH) were purchased from Alfa Aesar China (Shanghai, China). 3-ethoxy-4-methoxyphenol (EMP) was obtained from BOC Sciences (Shirley, NY, USA). 1-phenyl-1,2-ethanediol (PE) was bought from Acros Organics (NJ, USA). 2,5-dimethyl-4-hydroxy-3(2H)-furanone (DHF) was purchased from Tokyo Chemical Industry (Tokyo, Japan). N-(2-phenylethyl) acetamide (NPA) was kindly provided by Professor Helge Bode (Goethe University, Frankfurt, Germany). Phosphate-buffered saline (PBS, pH 7.4) was prepared with 100 mM of phosphate and 0.7% NaCl. Anticoagulant buffer (ACB, pH 4.5) was prepared with NaCl (186 mM), Na_2_EDTA (17 mM) and citric acid (41 mM).

### Fractionation of bacterial culture broth and TLC analysis

All bacterial isolates were cultured individually in TSB (1 L) at 28 °C with shaking at 180 rpm. After 48 h, bacterial pellets were separated from supernatant after centrifuging cultured broth at 10,000×*g* for 20 min at 4 °C. The resulting supernatant was subjected to fractionation of secondary metabolites by successively adding four different organic solvents (hexane, ethyl acetate, chloroform, and butanol) as described previously by Mollah et al. [35]. Resulting extracts were resuspended in methanol (0.4 mg/mL) and further diluted with DMSO or methanol to desired concentrations based on experimental purposes. During each fractionation step, the metabolite extraction was subjected to thin layer chromatography (TLC) by spotting it on a silica gel plate (20 × 20 cm: Merck, Darmstadt, Germany). A mixture of chloroform, methanol, and acetic acid (7:2.5:0.5, v/v) was used as an eluent. Spots in the silica gel plate were visualized and marked under a fluorescence analysis cabinet (Spectroline, CM-10, Westbury, NY, USA).

### Gas chromatography coupled with mass spectrophotometer (GC-MS) analysis

GC (7890B, Agilent Technologies, Santa Clara, CA, USA) equipped with MS (5977A Network, Agilent Technologies) was used for the prediction of the bacterial extracts in the methanol resuspension. It was performed as described by Mollah et al. [35]. An HP 5 MS column (non-polar column, Agilent Technologies) was used for GC. Helium was used as carrier gas at a flow rate of 1 mL/min. Split mode of injection with split ratio 10:1 was followed at 200 °C. The initial temperature was 100 °C for 3 min. It was raised to 300 °C at 5 °C/min. The oven temperature was maintained at 300 °C for 10 min. For recording mass spectra, EI mode at 70 eV with a scanning range of 33–550 m/z was used. Mass spectra were used for identification of purified samples using NIST11 database (U.S. Department of Commerce, Gaithersburg, MD, USA) and literature data (http://nistmassspeclibrary.com).

### Hemocyte-spreading behavior assay

Hemolymph (~ 250 μL) was collected from L5 larvae of *S. exigua* by cutting prolegs. It was mixed with 750 μL ACB. The hemolymph suspension was centrifuged at 800×*g* for 5 min and 800 μL supernatant was mixed with filter-sterilized 200 μL TC100 insect culture medium (Hyclone, Daegu, Korea). This hemocyte suspension (9 μL) and each bacterial metabolite (1 μL) were mounted on a glass slide to assess hemocyte-spreading behavior. For rescue experiment with AA (10 μg/μL in DMSO), 8 μL of cell suspension, 1 μL of bacterial metabolite, and 1 μL of AA were placed on a glass slide. After incubating the mixture at room temperature under darkness for 40 min, hemocytes were observed under a phase contrast microscope (Olympus) at 800 × magnification. As control, the dilution solvent (DMSO) of metabolites was used. Spread hemocytes were recognized based on cytoplasmic extension out of cell boundary. Each treatment was replicated three times with separately prepared suspension mixture. In each replication, 100 hemocytes were randomly chosen to assess hemocyte-spreading behavior.

### Nodulation assay

Hemocyte nodule formation was observed using 3 days old L5 larvae of *S. exigua* after injecting 3 μL of overnight grown *E. coli* Top 10 (10^4^ cells/larva) and 2 μL of DMSO to the hemocoel through prolegs using a microsyringe (Hamilton, Reno, NV, USA). To assess any inhibitory effect of bacterial extracts or secondary metabolites, 1 μL (1 μg/μL in DMSO) of test sample was co-injected with 3 μL of *E. coli* and 1 μL of DMSO. For AA rescue experiment, 1 μL of AA (10 μg/μL in DMSO) was co-injected with 1 μL of bacterial metabolite and 3 μL of *E. coli*. After incubation at 25 °C for 8 h, injected larvae were dissected to count melanized nodules under a stereomicroscope (Stemi SV11, Zeiss, Jena, Germany) at 50 × magnification. Each treatment was replicated three times using five larvae per replication.

### PLA_2_ enzyme assay

PLA_2_ activity was assessed using processes previously described by Vatanparast et al. [36]. Briefly, enzymes were extracted after grinding hemocytes of L5 larvae of *S. exigua* with PBS followed by centrifugation at 12,500×g for 5 min. The resulting supernatant containing the cytosol and membrane mixture was used as enzyme source. The reaction mixture (225 μL) contained 10 μL of PLA_2_ enzyme, 10 μL of Ellman’s reagent (5,5-di-thio-bis-(2-nitrobenzoicacid)), 5 μL of test inhibitor, and 200 μL of sPLA_2_ or cPLA_2_ substrate. DMSO was used instead of test inhibitor as control. Change in absorbance of reaction product was measured at wavelength of 405 nm using a microplate reader (Victor, PerkinElmer, Waltham, MA, USA). Each treatment was replicated three times with biologically independent samples.

### Bacterial pathogenicity test

To determine insecticidal activities of 14 bacterial isolates, bacterial cells after 24 h of culture were centrifuged at 10,000×*g* for 2 min at 4 °C. Cell pellet was re-suspended in PBS for injection. Bacterial suspension was diluted with PBS to obtain different concentrations (0, 10^1^, 10^2^, 10^3^, 10^4^, 10^5^ and 10^6^ colony-forming unit (cfu)/larva) and injected to hemocoel of L5 *S. exigua* larvae using a microsyringe. Before injection, larvae were surface sterilized with 70% ethanol. Bacterial dose was measured after plating the suspension onto LB agar medium followed by culturing at 28 °C for 48 h. Mortality was counted at 24 h after bacterial injection. As control bacterial treatment, *E. coli* was used. Injected larvae were incubated at room temperature with sufficient diet. Larvae were considered to be dead if there was no movement upon touching. Each treatment consisted of three replications using 10 larvae for each replication.

### Toxicity tests of bacterial metabolites and synthetic compounds

To determine toxicities of bacterial extracts and predicted metabolites, hemocoelic injection was performed. Dried bacterial extracts and metabolites were weighed and dissolved in DMSO to prepare different concentrations (0.001, 0.01, 0.1, 1 and 10 μg/μL) for toxicity assays. A 10 μL microsyringe was used for hemocoelic injection. Each test sample (3 μL) was injected to L5 larvae of *S. exigua*. DMSO was injected as control. Treated larvae were placed in 9 cm diameter dishes and incubated at room temperature. Mortality was determined every 24 h for 96 h after treatment. Larvae were considered as dead if there was no movement upon touching. Each test concentration was replicated three times using 10 larvae per replication.

### Statistical analysis

All assay data for continuous variables were subjected to one-way analysis of variance (ANOVA) using PROG GLM in SAS program [37]. For ANOVA, mortality data were subjected to arcsine transformation. Means were compared with the least significant difference (LSD) test at 0.05 level of Type I error. Median lethal dose (LD_50_) was calculated using EPA Probit Analysis Program, ver. 1.5 (U.S. Environmental Protection Agency, Washington, D.C., USA).

## Supplementary Information

**Additional file 1:** **Table S1.** Insecticidal activities of four organic extracts of culture broth of 14 entomopathogenic bacteria (EPB) of *Xenorhabdus* and *Photorhabdus* against L5 larvae of *S. exigua*. The bioassay was carried out by injecting 3 μL of bacterial culture broth extracts from different concentrations into the hemocoel of L5 larva. Each treatment dose used 10 larvae and each treatment replicated three times. **Table S2.** GC-MS prediction of secondary metabolites in organic extracts of bacterial culture broth from 14 species of *Xenorhabdus* and *Photorhabdus.* **Table S3.** Secondary metabolites predicted from *Photorhabdus temperata temperata* (Ptt), *Xenorhabdus hominickii* (Xh) and *Xenorhabdus ehlersii* (Xe) which were used for biological activity analysis. **Figure S1.** Chromatograms from the GC-MS analysis of the organic extracts of 14 bacterial culture broths for the prediction of bacterial metabolites. Four organic solvents such as hexane, ethyl acetate, chloroform and butanol were used to get organic extracts ‘HEX’, ‘EAX’, ‘CX’ and ‘BX’ from the bacteria, *Photorhabdus temperata* Subs*p. temperata* ANU101 (‘Ptt’), *Xenorhabdus hominickii* ANU101 (‘Xh’), *X. nematophila* K1 (‘XnK1’), *X. ehlersii* KSY (‘Xe’),, *X. nematophila* SK1 (‘XnSK1’), *X. nematophila* SK2 (‘XnSK2’), *Photorhabdus luminescens* KACC12123 (‘Pl 193’), *P. luminescens* subsp. *laumondii* KACC12283 (‘Pl laum’), *P. luminescens* subsp. 2 *thracensis* KACC12284 (‘Pl thra’), *X. nematophila* KACC12145 (‘Xn12145’), *X. nematophila* Mexico (‘XnM’), *X. nematophila* France (‘XnF’), *X. bovienii* (‘Xb’), and *X. poinarii* (‘Xp’). The extracts were dried using rotary evaporator and the resulting product was dissolved in methanol. After filtration, the sample was used for GC analysis and MS recording. NIST 11 database were used to predict compounds based on mass spectra. X axis indicates the retention time in min.

## Data Availability

All data generated or analysed during this study are included in this published article and its supplementary information.

## Abbreviations

AA: Arachidonic acid
ACB: Anticoagulant buffer
ANOVA: Analysis of variance
BA: Benzyl alcohol
BZA: Benzylideneacetone
BH: Benzeneethanol-4-hydroxy
CBA: Cyanobenzoic acid
CFU: Colony-forming unit
DHF: Dimethylhydroxyfuranone
DMSO: Dimethyl sulfoxide
EH: Ethyl-1-hexanol
EMP: Ethoxymethoxyphenol
IAAH: Indole-3-aceticacid hydrazide
IND: Indole
LD_50_: Median lethal dose
LSD: Least significant difference
MP: Mercaptophenol
MT: Mercaptobenzothiazole
NRPS: Non-ribosomal peptide synthetase
PC: Phosphatidylcholine
PKS: Polyketide synthase
PLA_2_: Phospholipase A_2_
Lrp: Leucine-responsive protein
NPA: N-(2-phenylethyl) acetamide
PE: Phenyl-1,2-ethanediol
PO: Phenoloxidase
Pl: *Photorhabdus luminescens*
Ptt: *Photorhabdus temperata temperata*
TPL: Tryptophol
TSB: Tryptic soy broth
Xb: *Xenorhabdus bovienii*
Xe: *Xenorhabdus ehlersii*
Xh: *Xenorhabdus hominickii*
Xn: *Xenorhabdus nematophila*
Xp: *Xenorhabdus poinarii*
