# Supplementary material for: Virulent secondary metabolites of entomopathogenic bacteria genera, Xenorhabdus and Photorhabdus, inhibit phospholipase A2 to suppress host insect immunity
Source: BMC Microbiol. 2020 Nov 23;20:359. doi: 10.1186/s12866-020-02042-9 (PMC7684946; doi:10.1186/s12866-020-02042-9)
Supplement: Supplementary file 1 — Additional file 1: Table S1. Insecticidal activities of four organic extracts of culture broth of 14 entomopathogenic bacteria (EPB) of Xenorhabdus and Photorhabdus against L5 larvae of S. exigua. The bioassay was carried out by injecting 3 μL of bacterial culture broth extracts from different concentrations into the hemocoel of L5 larva. Each treatment dose used 10 larvae and each treatment replicated three times. Table S2. GC-MS prediction of secondary metabolites in organic extracts of bacterial culture broth from 14 species of Xenorhabdus and Photorhabdus. Table S3. Secondary metabolites predicted from Photorhabdus temperata temperata (Ptt), Xenorhabdus hominickii (Xh) and Xenorhabdus ehlersii (Xe) which were used for biological activity analysis. Figure S1. Chromatograms from the GC-MS analysis of the organic extracts of 14 bacterial culture broths for the prediction of bacterial metabolites. Four organic solvents such as hexane, ethyl acetate, chloroform and butanol were used to get organic extracts ‘HEX’, ‘EAX’, ‘CX’ and ‘BX’ from the bacteria, Photorhabdus temperata Subsp. temperata ANU101 (‘Ptt’), Xenorhabdus hominickii ANU101 (‘Xh’), X. nematophila K1 (‘XnK1’), X. ehlersii KSY (‘Xe’),, X. nematophila SK1 (‘XnSK1’), X. nematophila SK2 (‘XnSK2’), Photorhabdus luminescens KACC12123 (‘Pl 193’), P. luminescens subsp. laumondii KACC12283 (‘Pl laum’), P. luminescens subsp. 2 thracensis KACC12284 (‘Pl thra’), X. nematophila KACC12145 (‘Xn12145’), X. nematophila Mexico (‘XnM’), X. nematophila France (‘XnF’), X. bovienii (‘Xb’), and X. poinarii (‘Xp’). The extracts were dried using rotary evaporator and the resulting product was dissolved in methanol. After filtration, the sample was used for GC analysis and MS recording. NIST 11 database were used to predict compounds based on mass spectra. X axis indicates the retention time in min. [file 12866_2020_2042_MOESM1_ESM.pdf]

Supplementary data

Table S1. Insecticidal activities of four organic extracts of culture broth of 14 entomopathogenic bacteria (EPB) of *Xenorhabdus* and *Photorhabdus* against L5 larvae of *S. exigua*. The bioassay was carried out by injecting 3 µL of bacterial culture broth extracts from different concentrations into the hemocoel of L5 larva. Each treatment dose used 10 larvae and each treatment replicated three times.

Table S2. GC-MS prediction of secondary metabolites in organic extracts of bacterial culture broth from 14 species of *Xenorhabdus* and *Photorhabdus.*

Table S3. Secondary metabolites predicted from *Photorhabdus temperata temperata* (Ptt), *Xenorhabdus hominickii* (Xh) and *Xenorhabdus ehlersii* (Xe) which were used for biological activity analysis.

Fig. S1. Chromatograms from the GC-MS analysis of the organic extracts of 14 bacterial culture broths for the prediction of bacterial metabolites. Four organic solvents such as hexane, ethyl acetate, chloroform and butanol were used to get organic extracts 'HEX', 'EAX', 'CX' and 'BX' from the bacteria, *Photorhabdus temperata* subsp. *temperata* ANU101 ('Ptt'), *Xenorhabdus hominickii* ANU101 ('Xh'), *X. nematophila* K1 ('XnK1'), *X. ehlersii* KSY ('Xe'), , *X. nematophila* SK1 ('XnSK1'), *X. nematophila* SK2 ('XnSK2'), *Photorhabdus luminescens* KACC12123 ('Pl 193'), *P. luminescens* subsp. *laumondii* KACC12283 ('Pl laum'), *P. luminescens* subsp.

thracensis KACC12284 (‘Pl thra’), *X. nematophila* KACC12145 (‘Xn12145’), *X. nematophila* Mexico (‘XnM’), *X. nematophila* France (‘XnF’), *X. bovienii* (‘Xb’), and *X. poinarii* (‘Xp’). The extracts were dried using rotary evaporator and the resulting product was dissolved in methanol. After filtration, the sample was used for GC analysis and MS recording. NIST 11 database were used to predict compounds based on mass spectra. X axis indicates the retention time in min.

Table S1. Insecticidal activities of four organic extracts of culture broth of 14 entomopathogenic bacteria (EPB) of *Xenorhabdus* and *Photorhabdus* against L5 larvae of *S. exigua*. The bioassay was carried out by injecting 3 µL of bacterial culture broth extracts from different concentrations into the hemocoel of L5 larva. Each treatment dose used 10 larvae and each treatment replicated three times.

**EPB**[1]	**Organic**[2] **extracts**	**LD_{50} (µg/larva) (95 % CI)**	**Slope ± SE**	**df**	**$\chi 2$**
Xn SK1	HEX	720.95 (355.7-1431.2)	0.30 ± 0.67	3	0.821
	EAX	399.45 (204.8-789.3)	0.35 ± 0.55	3	0.933
	CX	563.42 (290.3-1107.8)	0.33 ± 0.61	3	0.902
	BX	516.72 (253.8-1029.5)	0.42 ± 0.49	3	0.713
Xn SK2	HEX	432.42 (218.3-851.5)	0.35 ± 0.52	3	0.537
	EAX	205.59 (106.8-407.5)	0.42 ± 0.48	3	0.728
	CX	310.47 (157.9-608.9)	0.34 ± 0.58	3	0.899
	BX	248.91 (132.4-486.8)	0.39 ± 0.52	3	0.666
Xn M	HEX	305.57 (157.3-611.7)	0.33 ± 0.62	3	0.527
	EAX	259.14 (126.3-505.3)	0.38 ± 0.53	3	0.552
	CX	268.93 (132.6-522.8)	0.35 ± 0.59	3	0.554
	BX	228.91 (112.8-445.8)	0.41 ± 0.52	3	0.666
Xn F	HEX	278.78 (136.1-536.3)	0.33 ± 0.62	3	0.491
	EAX	184.31 (91.8-365.4)	0.43 ± 0.47	3	0.649
	CX	281.46 (140.3-563.2)	0.38 ± 0.54	3	0.526
	BX	195.58 (186.2-384.9)	0.41 ± 0.48	3	0.610
Xn 12145	HEX	522.41 (260.3-1021.3)	0.35 ±0.58	3	0.537
	EAX	372.19 (184.9-742.7)	0.38 ± 0.54	3	0.61
	CX	495.45 (252.7-969.8)	0.37 ± 0.56	3	0.592
	BX	383.88 (190.6-774.6)	0.37 ± 0.54	3	0.691
Xn K1	HEX	665.87 (332.8-1323.3)	0.35 ± 0.58	3	0.635
	EAX	492.56 (245.2-983.7)	0.39 ±0.53	3	0.732
	CX	617.69 (306.9-1221.6)	0.37 ± 0.55	3	0.688
	BX	512.57 (254.7-1018.7)	0.39 ±0.53	3	0.732
Xb	HEX	199.06 (101.1-401.2)	0.38 ± 0.54	3	0.862
	EAX	154.31 (77.9-302.5)	0.42 ± 0.47	3	0.649
	CX	203.77 (102.3-405.7)	0.42 ± 0.52	3	0.899
	BX	167.78 (85.3-332.4)	0.43 ± 0.48	3	0.937
Xe	HEX	787.58 (395.5-1572.1)	0.32 ± 0.64	3	0.952
	EAX	422.29 (214.5-841.8)	0.34 ± 0.59	3	0.988
	CX	629.09 (317.3-1254.2)	0.32 ± 0.63	3	0.942
	BX	584.94 (299.5-1162.9)	0.34 ± 0.52	3	0.963
Xh	HEX	219.32 (112.4-436.2)	0.33 ± 0.65	3	0.649
	EAX	132.24 (74.3-261.7)	0.37 ± 0.52	3	0.993
	CX	189.79 (98.6-367.4)	0.28 ± 0.68	3	0.971
	BX	92.44 (53.9-179.8)	0.33 ± 0.57	3	0.997
Xp	HEX	770.77 (387.2-1497.5)	0.28 ± 0.74	3	0.993
	EAX	483.92 (242.5-965.8)	0.29 ± 0.69	3	0.917
	CX	677.98 (341.61354.2)	0.29 ± 0.42	3	0.786
	BX	438.06 (221.7-875.1)	0.31 ± 0.56	3	0.929
Ptt	HEX	178.59 (95.3-376.2)	0.36 ± 0.56	3	0.967
	EAX	111.75 (57.1-120.9)	0.34 ± 0.61	3	0.945

	CX	167.21 (85.9-333.8)	0.32 ± 0.62	3	0.893
	BX	126.89 (65.4-245.5)	0.303 ± 0.66	3	0.961
Pl 193	HEX	350.37 (180.2-697.3)	0.32 ± 0.62	3	0.952
	EAX	209.94 (56.1-416.9)	0.31 ± 0.62	3	0.982
	CX	312.56 (158.5-621.4)	0.34 ± 0.59	3	0.932
	BX	242.99 (124.9-480.6)	0.32 ± 0.62	3	0.984
Pl laum	HEX	381.08 (190.7-754.9)	0.29 ± 0.71	3	0.931
	EAX	207.96 (106.7-412.8)	0.31 ± 0.62	3	0.985
	CX	301.41 (153.2-600.3)	0.30 ± 0.66	3	0.930
	BX	180.47 (93.5-357.8)	0.34 ± 0.58	3	0.897
Pl thra	HEX	536.96 (266.5-1067.4)	0.30 ± 0.66	3	0.961
	EAX	211.59 (109.2-413.7)	0.33 ± 0.59	3	0.883
	CX	314.34 (158.9-625.6)	0.38 ± 0.53	3	0.778
	BX	170.82 (88.4-336.2)	0.35 ± 0.58	3	0.972

[1] Entomopathogenic bacteria (EPB) acronyms are mentioned in Materials and Methods.
[2] Organic extract acronyms are: hexane extracts (‘HEX’), ethyl acetate extracts (‘EAX’), chloroform extract (‘CX’) and butanol extract (‘BX’).

Table S2. GC-MS prediction of secondary metabolites in organic extracts of bacterial culture broth from 14 bacterial isolates of *Xenorhabdus* and *Photorhabdus.*

RT[1]	Compound[2]	Xn SK1	Xn SK2	Xn M[3]	Xn F	Xn	Xn K1	Xb	Xe	Xh	Xp	Ptt	Pl 193	Pl	Pl thra
**2.246**	Ethanol, 2,2-diethoxy-		+												
**2.4**	4-Ethylamino-n-butylamine											+			
**2.453**	1-Butanamine, N-butyl-	+		+	+	+	+	+		+		+	+	+	+
**2.471**	Leucine		+												
**2.476**	n-Butylethylenediamine					+		+							
**2.5**	Phenol										+		+		+
**2.518**	1-Propanol, 3-(methylthio)-													+	+
**2.482**	Propanedioic acid, propyl-	+							+						
**2.848**	Glycerin												+		
**3.018**	1-Hexanol, 2-ethyl-									+					
**3.031**	Benzyl alcohol									+		+			
**3.191**	2,5-Dimethyl-4-hydroxy-3(2H)-furanone											+			
**3.628**	Pyrazine, 3-ethyl-2,5-dimethyl-		+							+					
**3.84**	Cyclobutene, 2-propenylidene-												+		+
**3.864**	Benzeneethanamine							+		+					+
**4.153**	Phenylethyl Alcohol				+				+	+	+	+	+	+	+
**4.602**	4H-Pyran-4-one, 2,3-dihydro-3,5-dihydroxy-6-methyl-	+		+	+	+	+	+	+	+		+	+	+	
**4.926**	Hexanoic acid, 5-oxo-, ethyl ester		+							+					
**4.997**	Octanoic acid			+	+	+	+	+				+			+
**5.045**	Benzeneacetic acid, methyl ester												+	+	+
**6.024**	Benzothiazole										+	+	+		
**6.621**	Benzeneacetic acid	+											+	+	+
**6.638**	1,1-Diisobutoxy-isobutane									+					
**6.768**	Butane, 1,1-dibutoxy-		+						+						
**7.17**	5-Thiazoleethanol, 4-methyl-			+	+	+	+	+		+	+	+			
**7.418**	Indole									+		+	+	+	+
**7.618**	Propanedioic acid, phenyl-														+
**7.79**	1,2-Ethanediol, 1-phenyl-											+			
**7.79**	Formamide, N, N-dibutyl-				+	+		+		+	+			+	
**8.652**	l-Leucine, N-methoxycarbonyl-, methyl ester						+								
**8.734**	Cyclohexasiloxane, dodecamethyl-									+					
**9.265**	Butanoic acid, butyl ester	+	+	+	+	+	+	+		+	+			+	+
**9.289**	Propanoic acid, 2-methyl-, 3-hydroxy-2,4,4-trimethylpentyl ester								+	+	+		+		
**9.903**	1-Tetradecene			+	+			+							+
**9.372**	n-Decanoic acid					+						+			
**9.431**	Propanoic acid, 2-methyl-, butyl ester							+		+					
**9.85**	Benzeneethanol, alpha.-(phenylmethyl)-												+	+	+

**9.915**	2-Tetradecene, (E)-			+	+			+		+			+		
**10.11**	Tetradecane			+	+	+	+	+	+	+	+		+		+
**10.375**	Benzeneethanol, 4-hydroxy-									+		+	+	+	+
**11.343**	Benzene, (2-isothiocyanatoethyl)-												+		
**11.349**	Phthalimide		+	+	+	+	+	+		+	+	+	+	+	+
**11.452**	o-Cyanobenzoic acid			+	+	+	+	+		+		+	+	+	+
**11.615**	Dodecane, 2-methyl-						+	+							
**11.798**	7-Methyl-octadecane						+								
**11.957**	Acetic acid, phenyl-, isopentyl ester													+	
**11.963**	Hexathiane								+		+		+		+
**12.264**	3-Ethoxy-4-Methoxyphenol									+					
**12.294**	Cyclopentadecane		+		+										
**12.335**	Acetamide, N-(2-phenylethyl)-									+		+			
**12.489**	Pentadecane	+	+	+	+	+	+	+	+		+	+	+	+	+
**12.489**	Phenol, 3,5-bis(1,1-dimethylethyl)-												+		
**12.654**	N-Butyryl-DL-homoserine lactone		+	+	+	+	+	+							
**12.672**	Phenol, 2,4-bis(1,1-dimethylethyl)-	+	+	+		+			+	+	+			+	+
**12.678**	Phenol, 2,5-bis(1,1-dimethylethyl)-				+		+	+							
**12.50**	Phenol, 2,6-bis(1,1-dimethylethyl)-											+			
**13.929**	Pentadecane, 2-methyl-		+				+								
**14.036**	Dodecanoic acid					+		+				+	+	+	+
**14.088**	Pentadecane, 3-methyl-		+		+		+								
**14.437**	2,6,10,14-Tetramethyl-7-(3-methylpent-4-enylidene) pentadecane		+												
**14.579**	Cetene	+	+		+	+		+			+		+	+	+
**14.59**	5-Octadecene, (E)-			+											
**14.602**	Z-8-Hexadecene			+	+		+		+			+			
**14.762**	Hexadecane	+	+	+	+	+	+	+	+		+	+	+	+	+
**14.885**	Cyclopropane, 1-methyl-1-(1-methylethyl)-2-nonyl-		+												
**14.938**	Indole-3-pyruvic acid									+		+			+
**14.962**	Tryptophol									+		+	+	+	+
**14.974**	1H-Indole-3-acetic acid, hydrazide									+		+			
**15.688**	7,9-Dimethyl-1,4-dioxa-7,9-diazacycloundecane-8-thione									+					
**15.741**	Methyl jasmonate												+		
**15.801**	Heptadecane, 3-methyl-						+								
**15.853**	2-Bromo dodecane		+												
**15.883**	Hexadecane, 7,9-dimethyl-						+								
**16.078**	2-Mercaptophenol									+		+			
**16.137**	Phenol, 2-[(1-methylethyl) thio]-							+							
**16.166**	Hexadecane, 2-methyl-		+		+		+								
**16.208**	1-Pentadecene											+			
**16.515**	4-Mercaptophenol		+											+	
**16.769**	Dodecane, 2,6,11-trimethyl-												+		
**16.969**	Heptadecane	+	+	+	+	+	+	+	+		+	+	+	+	+
**17.005**	Nonadecane, 9-methyl-				+										
**17.105**	Heptadecane, 2,6-dimethyl-		+							+					

**17.241**	N-Hexanoyl-DL-homoserine lactone		+												
**17.317**	1H-Benzimidazole, 2-(methylthio)-											+			
**17.395**	dl-Alanyl-l-leucine								+	+	+	+		+	+
**17.524**	1H-Benzimidazole, 2-(methylthio)-					+		+							
**17.589**	1H-Thieno[3,4-d] imidazole-4-propanoic acid, hexahydro-2-oxo-, [3aS-(3a.alpha.,4.beta.,6a.alpha.)]-			+											
**17.772**	Pyrrolo[1,2-a] pyrazine-1,4-dione, hexahydro-	+	+				+			+	+	+	+		+
**17.884**	Propanamide, 2-amino-3-(3-indolyl)-											+		+	
**18.008**	N-Isopropyl-N-propyl aminoethyl-2-chloride		+												
**18.203**	N, N-Dipropyltryptamine								+					+	
**18.469**	Heptadecane, 3-methyl-		+				+							+	
**18.345**	Cyclo-(glycyl-l-leucyl)	+	+		+				+			+			
**18.734**	5-Octadecene, (E)-		+										+		
**18.929**	1-Octadecene	+							+	+	+			+	+
**18.935**	E-15-Heptadecenal		+			+	+	+							
**19.077**	Octadecane	+	+	+	+	+	+	+	+		+	+	+	+	+
**19.106**	Hexadecane, 7,9-dimethyl-											+			
**19.106**	1H-Indole-3-acetic acid, methyl ester									+			+		+
**19.201**	1-Hexadecanol, 2-methyl-											+			
**19.231**	D, L-3-Camphorcarboxylic acid					+									
**19.277**	Hexadecane, 2,6,10,14-tetramethyl-		+												
**19.289**	Dicyclohexyldisulphide										+	+	+		
**19.756**	Phenol, 3,5-dimethoxy-	+	+	+				+	+	+	+		+	+	+
**19.773**	1H-Indene, 2-butyl-5-hexyloctahydro-									+					
**20.34**	2,6-Dichlorobenzenesulfonyl chloride						+								
**20.352**	Benzene, 1,2-bis(1-buten-3-yl)-					+									
**20.517**	Octadecane, 3-methyl-		+												
**20.623**	1-Nonadecene	+	+	+	+	+	+				+	+	+	+	
**20.671**	Cyclotetradecane			+											
**20.677**	1H-Indole-3-ethanol, acetate (ester)									+		+		+	
**20.718**	4-Cyclopenta-1,3-dienylbutyric acid, ethyl ester						+								
**20.883**	Hexadecane, 1-chloro-														+
**20.99**	Benzeneacetic acid, 2-phenylethyl ester												+		+
**21.019**	Propanamide, 2,2,3,3,3-pentafluoro-N-(2-phenylethyl)-											+		+	
**21.102**	E-8-Methyl-9-tetradecen-1-ol acetate				+										
**21.09**	Nonadecane	+	+				+						+	+	
**21.131**	2-Dodecen-1-yl (-) succinic anhydride									+	+	+	+		
**21.131**	1,13-Tetradecadien-3-one											+			

**21.35**	L-Proline, N-valeryl-, decyl ester									+			+		
**21.349**	Pentadecanoic acid, 13-methyl-, methyl ester														+
**21.503**	Pyrrolo[1,2-a] pyrazine-1,4-dione, hexahydro-3-(2-methylpropyl)-	+	+	+	+	+	+	+		+		+	+	+	+
**21.55**	Hexadecanoic acid, methyl ester		+								+	+	+	+	
**21.751**	2,5-Furandione, 3-dodecyl-								+				+		
**21.804**	Benzenepropanoic acid, 3,5-bis(1,1-dimethylethyl)-4-hydroxy-, methyl ester	+		+	+	+	+	+	+	+	+	+	+	+	+
**21.969**	Stannane, tetraethyl-									+					
**22.005**	2',4'-Dihydroxyacetophenone oxime					+									
**22.146**	1,2-Benzenedicarboxylic acid, butyl 2-methylpropyl ester		+												
**22.147**	2-Mercaptobenzothiazole				+	+	+		+	+		+			
**22.158**	1,2-Benzenedicarboxylic acid, bis(2-methylpropyl) ester							+							
**22.182**	Diethyl Phthalate					+					+	+		+	
**22.701**	E-15-Heptadecenal											+		+	+
**22.731**	3-Phenylbicyclo (3.2.2) nona-3,6-dien-2-one											+			
**22.778**	Cyclohexane, 1-(1,5-dimethylhexyl)-4-(4-methylpentyl)-		+												
**22.885**	Cyclohexadecane, 1,2-diethyl-	+	+			+		+							
**23.009**	Eicosane	+	+	+	+	+	+	+	+		+	+	+	+	+
**23.032**	Octadecane, 1-chloro-						+		+						
**23.144**	Cyclic octaatomic sulfur			+	+	+	+	+	+	+	+		+	+	+
**24.03**	E-11-Methyl-12-tetradecen-1-ol acetate											+	+		
**24.26**	Fluorene, 4-[1,2-dihydroxyethyl]-					+				+					
**24.419**	Oleanitrile	+	+	+	+	+	+	+	+		+	+			+
**24.496**	2-Benzimidazolethiol				+		+								
**24.65**	2-Methyl-E-7-octadecene											+		+	
**24.85**	Heptadecanenitrile		+												
**24.886**	1-Eicosene			+			+	+		+					
**24.886**	Nonadecanenitrile				+					+					
**24.891**	Octadecanenitrile					+		+			+	+			+
**24.992**	DL-Tryptophan, N-acetyl-													+	+
**25.104**	Indole-3-butyramide													+	
**25.328**	Heptadecanoic acid, 16-methyl-, methyl ester			+					+						
**25.329**	Heptadecanoic acid, 14-methyl-, methyl ester						+			+					
**25.411**	2,5-Piperazinedione, 3-methyl-6-(phenylmethyl)-	+	+		+		+	+	+	+	+	+	+		+
**25.777**	Alloaromadendrene	+													
**25.813**	2,5-Piperazinedione, 3-(phenylmethyl)-	+	+							+		+			
**25.854**	Androstenedione												+		
**26.32**	Z-5-Nonadecene										+				+

**26.403**	E-8-Methyl-9-tetradecen-1-ol acetate									+					+
**26.479**	9-Tricosene, (Z)-*			+											
**26.509**	1-Docosene	+			+	+	+	+	+	+					
**26.604**	Octadecane, 2,6,10,14-tetramethyl-							+							
**26.639**	Docosane			+	+		+	+	+	+	+		+	+	+
**26.94**	Diethyldithiophosphinic acid	+	+	+	+	+	+	+	+	+	+				
**27.076**	Pyrene, 4-methyl-											+			
**27.507**	2,5-Piperazinedione, 3-benzyl-6-isopropyl-		+	+	+	+	+	+	+	+	+				
**29.302**	Pyrrolo[1,2-a] pyrazine-1,4-dione, hexahydro-3-(phenylmethyl)-	+	+	+	+	+	+	+		+		+	+	+	+
**28.817**	Cyclo-(l-leucyl-l-phenylalanyl)	+			+	+	+		+	+		+	+	+	+
**28.912**	Benzyl butyl phthalate		+												
**29.16**	9-Octadecenamide, (Z)-	+	+	+		+		+				+	+		
**29.827**	1-Docosanethiol							+							
**31.061**	3-Benzylidene-hexahydro-pyrrolo[1,2-a] pyrazin-1,4-dione						+								
**31.078**	Hexanoic acid, 2-ethyl-, hexadecyl ester		+												
**32.153**	Bis(2-ethylhexyl) phthalate	+	+	+	+	+	+	+	+	+	+	+	+	+	+
**32.159**	Diisooctyl phthalate	+													
**32.212**	Di-n-octyl phthalate		+									+			
**32.424**	Pentanamide, N-[2-(indol-3-yl)] ethyl-									+					+
**32.454**	Acetamide, N-[2-(1H-indol-3-yl) ethyl]-													+	
**32.755**	Cyclotrisiloxane, hexamethyl-							+	+	+		+		+	+
**33.026**	Zinc,bis(dimethylcarbamodithioato-S,S')-, (T-4)-											+	+		
**34.709**	l-Proline, N-allyloxycarbonyl-, octadecyl ester				+										
**35.488**	4-(cis-6-Methoxymethyl-3,4-dimethyl-3-cyclohexenyl)-trans-3-buten-2-one 2,4-dinitrophenylhydrazone					+									
**35.813**	5-Methyl-2-phenylindolizine					+									
**44.196**	Zinc dibutyldithiocarbamate											+			
	Σ182	32	53	36	44	44	49	47	33	60	36	63	54	49	53

[1] Retention time at GC analysis.
[2] Compounds are predicted from four organic extracts of bacterial culture broths.
[3] Bacterial acronyms are described in Materials and Methods.

Table S3. Secondary metabolites predicted from *P. temperata temperata*, *Xenorhabdus hominickii*, and *X. ehlersii*

Compounds (Acronym)	Structure
Benzyl alcohol (BA)	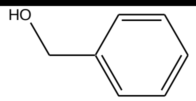
Indole (IND)	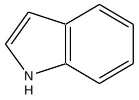
Benzeneethanol, 4-hydroxy- (BH)	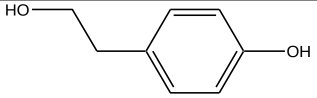
o-Cyanobenzoic acid (CBA)	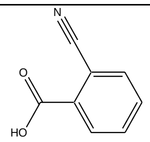
Acetamide, N-(2-phenylethyl)- (NPA)	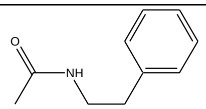
Tryptophol (TPL)	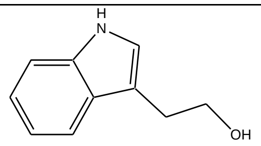
1H-Indole-3-acetic acid, hydrazide (IAAH)	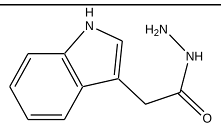
2-Mercaptophenol (MP)	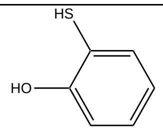
2-Mercaptobenzothiazole (MT)	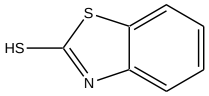

1-Hexanol, 2-ethyl- (EH)	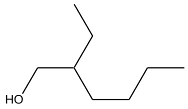
3-Ethoxy-4-Methoxyphenol (EMP)	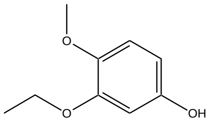
2,5-Dimethyl-4-hydroxy-3(2H)-furanone (DHF)	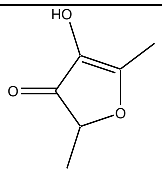
1,2-Ethanediol, 1-phenyl- (PE)	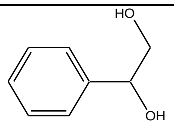

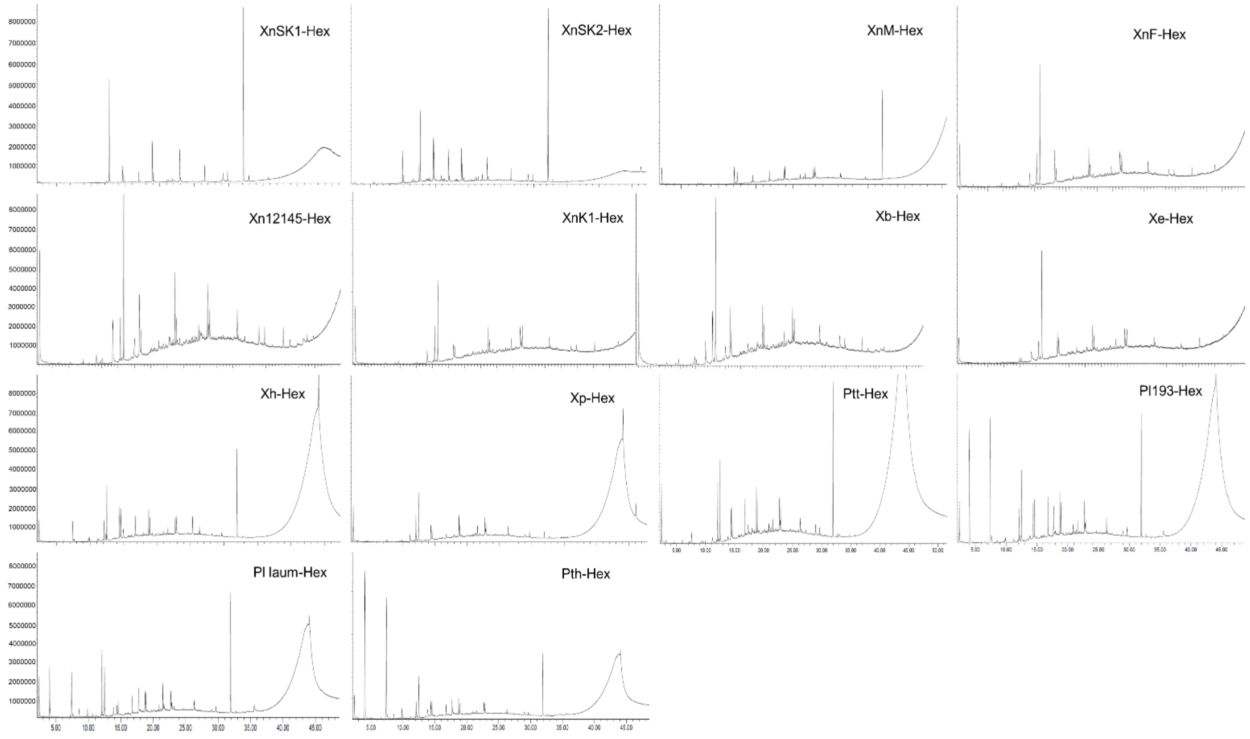
XnSK1-Hex
XnSK2-Hex
XnM-Hex
XnF-Hex
Xn12145-Hex
XnK1-Hex
Xb-Hex
Xe-Hex
Xh-Hex
Xp-Hex
Ptt-Hex
Pl193-Hex
Pl laum-Hex
Pth-Hex

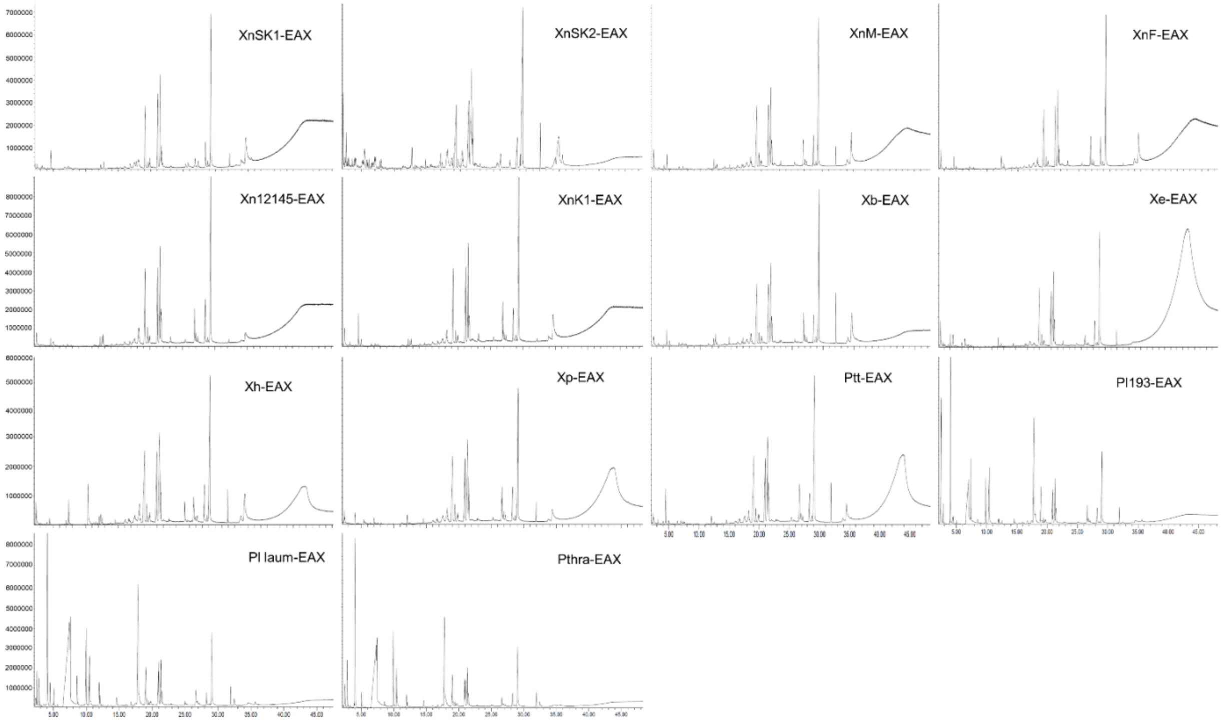
XnSK1-EAX
XnSK2-EAX
XnM-EAX
XnF-EAX
Xn12145-EAX
XnK1-EAX
Xb-EAX
Xe-EAX
Xh-EAX
Xp-EAX
Ptt-EAX
Pl193-EAX
Pl laum-EAX
Pthra-EAX

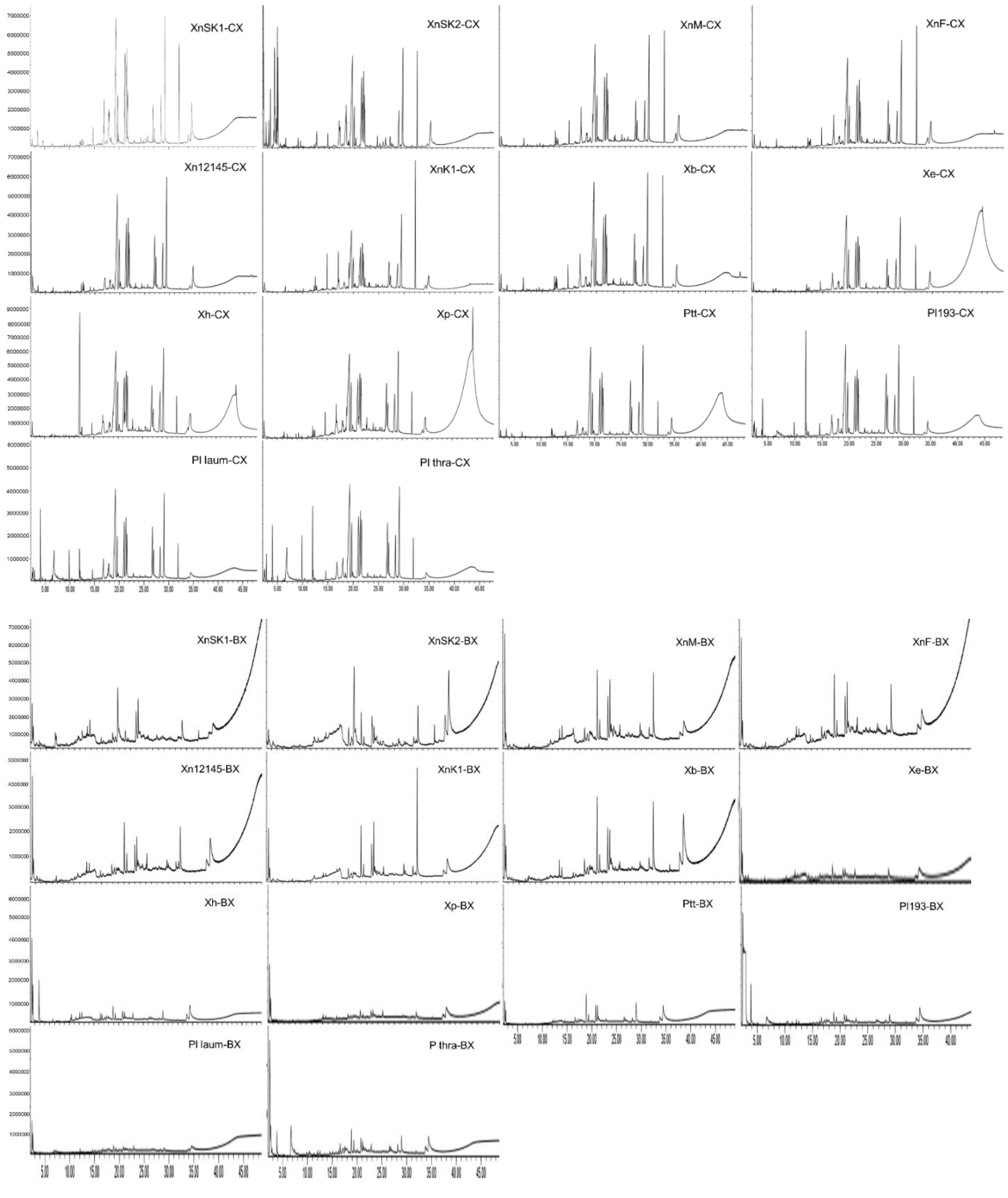
XnSK1-CX
XnSK2-CX
XnM-CX
XnF-CX
Xn12145-CX
XnK1-CX
Xb-CX
Xe-CX
Xh-CX
Xp-CX
Ptt-CX
Pl193-CX
Pl laum-CX
Pl thra-CX
XnSK1-BX
XnSK2-BX
XnM-BX
XnF-BX
Xn12145-BX
XnK1-BX
Xb-BX
Xe-BX
Xh-BX
Xp-BX
Ptt-BX
Pl193-BX
Pl laum-BX
P thra-BX

Fig. S1
